# Predictive Feasibility of the Graz Malnutrition Screening, Controlling Nutritional Status Score, Geriatric Nutritional Risk Index, and Prognostic Nutritional Index for Postoperative Long-Term Mortality After Surgically Treated Proximal Femur Fracture

**DOI:** 10.3390/nu16244280

**Published:** 2024-12-11

**Authors:** Domenik Popp, Marie Stich-Regner, Lukas Schmoelz, Sara Silvaieh, Stephan Heisinger, Arastoo Nia

**Affiliations:** 1 Clinical Division of Traumatology, Department of Orthopedics and Trauma Surgery, Medical University of Vienna, 1090 Vienna, Austria; domenik.popp@meduniwien.ac.at (D.P.); n1542281@students.meduniwien.ac.at (M.S.-R.); lukas.schmoelz@meduniwien.ac.at (L.S.); stephan.heisinger@meduniwien.ac.at (S.H.); 2 Department of Orthopedics and Traumatology, University Clinic Neunkirchen, 1090 Vienna, Austria; 3 Department of Neurology, Medical University of Vienna, 1090 Vienna, Austria; sara.silvaieh@meduniwien.ac.at

**Keywords:** hip fracture, risk prediction, mortality, GMS, PNI, GNRI, CONUT, malnutrition

## Abstract

Background: Hip fractures are a prevalent and serious health issue, particularly among the elderly population aged >65 years. These injuries are associated with elevated rates of postoperative complications and mortality, significantly diminishing patients’ quality of life in both the short- and long-term. The prognosis for recovery is further exacerbated in individuals with signs of malnutrition. The primary objective of this study was to evaluate the predictive value of four distinct nutritional assessment scores in relation to postoperative mortality in patients undergoing surgical intervention for hip fractures at 1, 3, 6, 12, and 24 months. Methods: This observational study included patients admitted to the Department of Traumatology at the Medical University for the surgical management of hip fractures between January 2019 and November 2021. Nutritional assessment scores were derived from a retrospective analysis of clinical data. The statistical correlation between nutritional scores and postoperative mortality outcomes was rigorously evaluated. Results: Logistic regression analysis revealed a statistically significant correlation (*p* < 0.01) between all four nutritional scores and postoperative mortality risk. The malnourished cohorts demonstrated a markedly higher risk of mortality compared to those with adequate nutritional status, as indicated by the following risk ratios: Graz Malnutrition Screening (risk ratio = 2.53–1.68), Prognostic Nutritional Index (risk ratio = 2.44–1.74), Geriatric Nutritional Risk Index (risk ratio = 2.05–1.58), and Controlling Nutritional Status (risk ratio = 2.34–1.46). Despite these findings, the receiver operating characteristic analysis yielded area under the curve (AUC) values ranging from 0.64 to 0.68, indicating limited predictive power. Conclusions: Although a significant correlation existed between the evaluated nutritional scores and postoperative mortality, the predictive value of these scores was quantitatively low. No single nutritional assessment tool has emerged as a strong predictor of postoperative outcomes in this patient population. Consequently, implementation of any specific nutritional screening tool for standard assessment in patients with hip fractures is not recommended at this time. Nevertheless, given the established association between malnutrition and postoperative mortality, a comprehensive evaluation of nutritional status is advisable and further research is needed.

## 1. Introduction

As global life expectancy increases and the population ages, the incidence of hip fractures has become a significant and growing challenge for healthcare systems. While hip fractures are rare among younger adults, where they are typically the result of high-energy trauma (e.g., motor vehicle accidents), they occur much more frequently in the elderly because of low-energy events, such as a simple fall from standing height [1,2,3]. The vast majority of affected individuals (approximately 70%) are women, with an average age of 80 years [4,5]. This demographic shift underscores the increasing vulnerability of older adults who are predisposed to fractures due to osteoporosis, frailty, and other age-related conditions.

The economic and social burden associated with hip fractures is substantial. Healthcare costs related to acute treatment, rehabilitation, and long-term care are notably high [6,7,8]. Moreover, the personal impact on patients is often severe, with a significant loss of mobility, independence, and overall quality of life. More than half of the patients fail to regain their pre-injury mobility within a year after the fracture, and a large proportion becomes permanently dependent on external assistance [9,10]. One month post-injury, mortality rates range from 7–10%, with figures rising to 20% by six months [11]. Despite advancements in surgical techniques and postoperative care, the one-year mortality rate remains elevated, ranging from 26% to 36%, depending on the study [12]. Mortality risks are especially heightened in patients who experience a second hip fracture within the same year [13,14].

Nutritional status is a key modifiable factor that influences the postoperative outcomes in this vulnerable population. Malnutrition is prevalent among geriatric patients, particularly those hospitalized with acute injuries, such as hip fractures, and is associated with extended hospital stays, delayed rehabilitation, and increased mortality [12]. Surgical interventions in older adults trigger an inflammatory and metabolic stress response, resulting in enhanced catabolism and muscle loss, which are particularly detrimental in elderly patients due to pre-existing frailty and reduced muscle mass [15]. Malnutrition exacerbates these challenges and significantly worsens the recovery process [16,17].

Given the often subtle and under-recognized nature of malnutrition in older adults, routine nutritional screening is essential. Several studies have demonstrated that malnutrition significantly influences postoperative outcomes across various patient groups, with a reported prevalence of 20–50% among hospitalized patients [18,19]. In Austria, Schönherr et al. [20] reported a malnutrition prevalence of up to 26.2% among patients in hospitals and care homes and malnutrition rates as high as 50% in patients undergoing joint replacement [21].

Nutritional assessment tools provide a cost-effective and practical means of identifying patients at risk for poor outcomes [19,22]. This study evaluated the feasibility of four nutritional screening scores for assessing postoperative mortality in patients with surgically treated proximal femur fractures. The early identification of high-risk patients enables the implementation of targeted interventions, potentially improving survival rates and optimizing recovery outcomes.

## 2. Materials and Methods

### 2.1. Study Design and Population

This retrospective cohort study included patients aged >60 years who sustained a proximal femur fracture and underwent surgical treatment at the Division of Traumatology, Department of Orthopedics and Traumatology, Medical University of Vienna, between January 2019 and November 2021. A total of 1751 patients were initially screened; after applying the exclusion criteria, 1500 patients were enrolled. Patients with insufficient data, periprosthetic or pathological fractures, polytrauma, or conservative treatment were excluded.

The cohort included 70.8% women (n = 1062), with an overall mean age of 81.1 years (range: 59–106). The body mass index (BMI) was calculated for all patients, with a mean BMI of 23.8 kg/m^2^ (Min: 16.9; Max 45.8; SD: ±0.6). According to WHO guidelines, a BMI below 18.5 kg/m^2^ is classified as malnutrition [23]. Mortality data, including the month and year of death, were retrieved from the Austrian Death Register on 30 April 2024. Patients absent from the registry during the study period were presumed to be alive.

### 2.2. Nutritional Screening Tools

Four nutritional screening tools were assessed to evaluate their ability to predict postoperative mortality at 1, 3, 6, 12, and 24 months.

Graz Malnutrition Screening (GMS): Developed at the University Hospital Graz, GMS evaluates weight loss, BMI, reduced food intake, and disease severity, with an additional point for age >65 years. A score ≥3 indicates a risk of malnutrition [24].

Geriatric Nutritional Risk Index (GNRI): A refinement of the Nutritional Risk Index (NRI) tailored for older patients. The GNRI calculates nutritional risk based on albumin levels and body weight, categorizing patients into four risk groups: major risk (<82), moderate risk (82–<92), low risk (92–≤98), and no risk (>98) [25].

Prognostic Nutritional Index (PNI): Initially developed by Buzby et al. [26], the PNI uses serum albumin level and total lymphocyte count to assess both nutritional and immunological status. Lower PNI scores (<40) were associated with a poor prognosis and higher surgical risk.

Controlling Nutritional Status (CONUT): Developed by the Hospital Universitario de la Princesa, Madrid, CONUT evaluates serum albumin, cholesterol, and lymphocyte count. It provides a comprehensive assessment of protein reserves, caloric depletion, and immune functions [27].

### 2.3. Surgical Treatment

Surgical interventions followed established guidelines based on the fracture type. Intracapsular fractures (medial and lateral femoral necks) were treated with cannulated screws, dynamic hip screws, hemiarthroplasty, or total arthroplasty. Extracapsular fractures (pertrochanteric and subtrochanteric) were treated predominantly with intramedullary nailing with dynamic hip screws used in select cases.

### 2.4. Statistical Analysis

Preoperative data, including nutritional parameters, were collected from routine clinical assessments performed on the day of surgery or the day prior to surgery. The predictive abilities of the four nutritional screening tools were evaluated using the following statistical methods.

▪ Categorical variables were presented as counts and percentages, while continuous variables were reported as means with standard deviations or medians with interquartile ranges, depending on normality (assessed using the Shapiro–Wilk test).
▪ Associations between nutritional scores and postoperative mortality were analyzed using the chi-squared test and logistic regression analysis.
▪ Receiver operating characteristic (ROC) curves and area under the curve (AUC) were calculated to determine the discriminative ability of each score for predicting mortality at 1, 3, 6, 12, and 24 months. An AUC value of 0.5 served as a threshold for stratifying patients into “deceased” and “alive” groups, with those exceeding a predicted mortality rate of 50% classified as “more likely to die”.

Data analyses were performed using the SPSS software (v.21, IBM Corp., Armonk, NY, USA), with statistical significance set at *p* < 0.05.

## 3. Results

The mean BMI of the study cohort was 23.82 kg/m^2^. Among the 1500 participants, 129 (8.6%) were classified as undernourished according to the WHO criteria [28]. Normal weight was observed in 868 patients (57.8%), and 384 (25.6%) were classified as overweight. Furthermore, 96 patients (6.4%) had Class I obesity, 19 (1,3%) had Class II obesity, and 4 (0.3%) had Class III obesity.

Mortality data were retrieved on 21 December 2023. In the first postoperative month, 81 patients (5.4%) died. By three months post-surgery, 165 patients had died, resulting in a three-month mortality rate of 11%. Six months after surgery, 225 patients died, yielding a six-month mortality rate of 15%. By 12 months, 321 patients had died, corresponding to a one-year mortality rate of 21.4% (Table 1). At the end of the observation period, 473 of the 1500 patients died, leading to an overall mortality rate of 31.5% at 24 months postoperatively.

### 3.1. GMS

The GMS revealed that the majority of patients (41.9%) scored two points. Scores of 0 (2.1%) and between 5–8 points (6.5% combined) were rare. A significant proportion of the patients achieved scores of 1 (21.1%), 3 (17.4%), or 4 (11%). Consequently, approximately 34.9% of patients met the GMS criteria for malnutrition.

**Figure 1:**
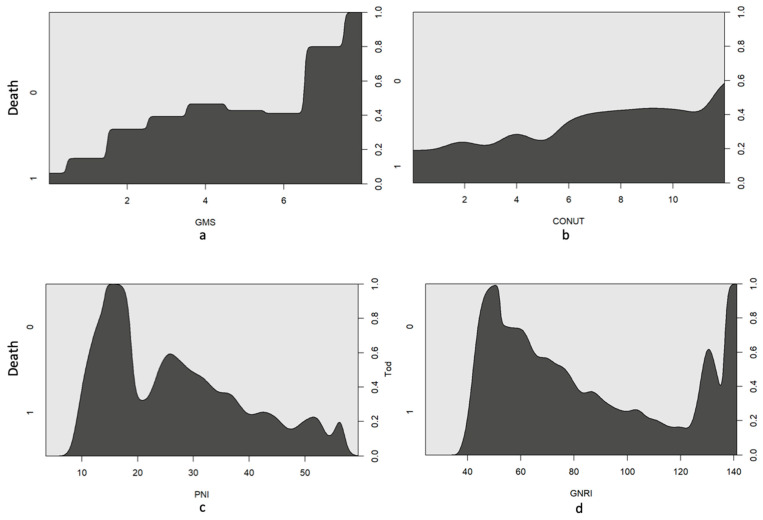
(**a**–**d**): Conditional density plots of the used screening tools including cut-off values for mortality prediction.

As illustrated in Figure 1a, the survival probability decreased with increasing GMS scores. The highest mortality rate was observed in the subgroup with GMS scores of 7–8, although this group represented only a small fraction of the overall population (12 patients). Logistic regression analysis yielded *p*-values of <0.01 at all time points, indicating a significant association between postoperative mortality and GMS scores.

This association was further substantiated by the risk ratios presented in Table 2. One month postoperatively, the mortality risk for patients with a GMS score of 3 or higher was 2.35 times greater than that for those with scores below 3. Although this relative risk decreased over the follow-up period, it remained significantly elevated. At the conclusion of the observation period, the mortality risk among malnourished patients based on the GMS criteria was 1.68 times higher compared to that among malnourished patients classified as having normal nutritional status.

### 3.2. CONUT

As shown in Table 3, only 84 patients (5.6%) exhibited a normal nutritional status (CONUT 0–1). A total of 548 patients (36.6%) presented with a mild nutritional risk (CONUT 2–4). The largest cohort, comprising 698 patients (46.6%), demonstrated a moderate nutritional risk (CONUT 5–8), while 170 patients (11.2%) were classified as having a severe nutritional risk (CONUT 9–12).

The number of deceased patients increased in direct proportion to the CONUT score, with higher scores correlating with higher mortality during the observation period (Figure 1b). Logistic regression confirmed significant *p*-values of <0.01, reinforcing the association between postoperative mortality and CONUT scores. A cut-off value of 5 was established for risk ratios, contrasting patients with moderate to severe nutritional risk with those with mild or no nutritional risk. Table 2 lists the risk ratios at various time points.

Notably, the risk of mortality was greater during the first three postoperative months than during the first month alone.

### 3.3. PNI

More than 75% of the patients had PNI values below 45, indicating an elevated postoperative risk. Specifically, 60.7% of the patients had PNI values below 40, indicating an unfavorable prognosis (Figure 1c). Only 15.2% of the patients exhibited normal nutritional status based on the PNI criteria. The maximum recorded PNI was 59.6, with a minimum value of 3.5.

A lower PNI was associated with an increased mortality rate, suggesting an inverse relationship between mortality and PNI scores. Notably, patients with PNI values < 20 exhibited a significantly elevated mortality rate, albeit with a limited sample size (8 patients). Logistic regression yielded *p*-values < 0.01, reaffirming the relationship between postoperative mortality and PNI values.

For patients with a PNI score < 40, the mortality risk in the first postoperative month was 2.41 times greater compared to those with higher PNI scores. Similar to GMS and CONUT, the risk of mortality during the first three postoperative months exceeded that of the first month alone (Table 2).

### 3.4. GNRI

Both the median and mean GNRI were 93.6, indicating that >50% of patients experienced no or only mild nutritional risk. Moderate nutritional risk was identified in 28.4% of the patients, while 15.5% exhibited a significant nutritional risk. The minimum recorded GNRI was 24, with a maximum of 141.3. Importantly, both extremely high and low GNRI values were associated with elevated mortality rates (Figure 1d). However, it is crucial to consider the limited sample size at both values. The mortality rate was inversely proportional to the GNRI levels.

Logistic regression analysis confirmed *p*-values of <0.01, substantiating the link between postoperative mortality and GNRI scores. Risk ratios for GNRI values below 92 indicated a 1.96 times higher mortality risk in the first postoperative month for patients with moderate or significant nutritional risk than for those with minor or no nutritional risk (Table 2). As observed with other nutritional scores, the relative risk continued to rise during the first three months postoperatively before tapering off, remaining 1.58 times higher by the end of the observation period.

### 3.5. Predictive Power of Nutritional Scores

Nutritional scores were evaluated using ROC curves to determine their predictive power. This forecasting model compares the true positive rate (sensitivity) against the false positive rate (1—specificity). In this context, it involves contrasting accurately predicted deaths due to poor nutritional status based on the respective scores with deaths that were not predicted. The AUC can range from 0 to 1, where a value of 1.0 indicates perfect predictions and all forecasts are correct with no false positives. An AUC of 0.5 represents a random classifier is equivalent to chance, indicating no predictive power. AUC values between 0.5 and 0 suggest that the chosen model may yield results contrary to expectations.

**Figure 2:**
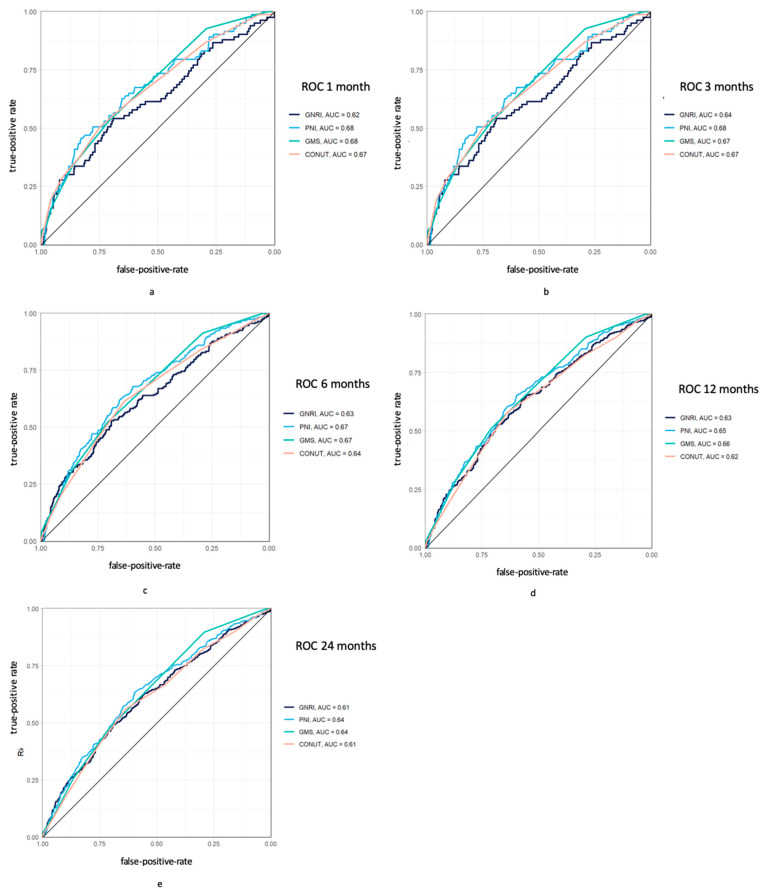
(**a**–**e**): AUC for mortality-prediction at 1, 3, 6, 12, and 24 months.

In the first postoperative month, the interpretation of the ROC curves for all nutritional scores yielded similar and modestly positive results. Only the Geriatric Nutritional Risk Index (GNRI) exhibited a slightly lower AUC. The AUC values ranged between 0.62 and 0.68, indicating moderate predictive power (Figure 2a).

At 90 days postoperatively, the predictive capacity of the GNRI improved marginally, whereas the predictive strength of the other nutritional scores remained relatively stable. Overall, the predictive power was moderate (Figure 2b).

By 180 days (6 months) post-surgery, the AUC for nearly all nutritional scores had decreased, with the GMS remaining stable. However, its predictive power remained low (Figure 2c). One year after surgical intervention, the AUC values were either consistent or slightly reduced. The predictive capacity remained low (Figure 2d).

At the end of the observation period, all nutritional scores had the lowest AUC values (Figure 2e). Although the predictive power appeared to decrease over time, the differences were marginal and likely insignificant. Consequently, overall predictive capacity remained low throughout the observation period.

## 4. Discussion

### 4.1. Nutritional Scores

Despite years of research on nutritional screening, no single nutritional score has emerged as the gold standard [29,30]. One significant reason for this is that none of the available screening tools consistently yield reliable results across all metabolic conditions [31]. Dent et al. [30] reviewed various nutritional scores, including the Subjective Global Assessment (SGA), Mini Nutritional Assessment (MNA), Mini Nutritional Assessment-Short Form (MNA-sf), Malnutrition Universal Screening Tool (MUST), Simplified Nutritional Appetite Questionnaire (SNAQ), and Geriatric Nutritional Risk Index (GNRI), and concluded that each serves as a useful instrument for the early detection of malnutrition in older patients, but only in conjunction with a clinical nutritional assessment does they provide reliable outcomes. Thus, evaluation of nutritional scores within patient populations with specific disease profiles is warranted.

Pratt et al. [32] investigated the impact of improved care for malnourished patients and those at risk of malnutrition in a study conducted at Tampa General Hospital, a large teaching hospital in the United States. Through the establishment of interdisciplinary teams, identification of care gaps, and enhanced involvement of clinical nutritional management, they demonstrated a 25% reduction in the length of hospital stay and a 35.7% decrease in infection rates among the patients studied [32]. Reliable malnutrition screening is essential for the effective implementation of such interventions.

While each nutritional score examined in this study correlated significantly with postoperative mortality, the predictive power—both individually and in combination as a predictive model—remains low. Numerous studies have described the statistical associations between nutritional scores and postoperative mortality; however, research focusing specifically on their predictive capacity is limited. This low predictive value arises from the high sensitivity of these scores for malnutrition screening, which contrasts with their moderate specificity for mortality.

### 4.2. GMS

The GMS has the least extensive literature compared with the other nutritional scores assessed in this study. The GMS was originally validated against Nutritional Risk Screening (NRS) and MNA-sf [32]. Traub et al. [33] found in 2020 that the GMS could not be validated for use in patients with liver cirrhosis. Currently, there is no available literature examining GMS in the context of hip joint-related fractures.

Statistical analyses yielded promising results, indicating a degree of validity in the studied patient cohort; however, further research is necessary to make definitive conclusions. Unlike the other screening instruments evaluated, the GMS incorporates patient-reported data on appetite, weight loss, and pre-existing conditions, as interpreted and documented by attending medical and nursing teams. This approach offers advantages through potential clinical evaluation during data collection but also increases the risk of observer bias and the associated time burden.

### 4.3. CONUT

The CONUT score, derived from predefined, automated evaluations of the total lymphocyte count, serum albumin concentration, and total cholesterol levels, minimizes the risk of observer bias. Additionally, the fact that patients do not need to provide self-reports is advantageous, particularly given that many may suffer from cognitive impairments, such as dementia.

However, it is crucial to note that reliable assessments of nutritional status can only be performed in conjunction with clinical evaluation [30]. CONUT score has been linked to postoperative outcomes in various oncological conditions [34,35]. Studies by Yagi et al. [35] and Kotera et al. [25] investigated CONUT scores in patients with hip fractures.

Yagi et al. [35] conducted a retrospective analysis of 211 patients over 50 years old who underwent surgery for hip fractures from 2013 to 2018, classifying a CONUT score > 1 as malnourished. Their findings indicated a malnutrition rate of 78.7% in the study population. In our analysis, 94.6% of the patients achieved a CONUT score > 1 and were further stratified into three severity categories [27]. However, Yagi et al. [35] did not assess the specificity. While a high preoperative CONUT score was correlated with increased postoperative complication rates (odds ratio: 1.21), no predictive value was established for CONUT as a screening instrument [35].

Kotera et al. [25] evaluated 607 patients with hip fractures at a Japanese Hospital between 2012 and 2018. They also retrospectively computed the necessary findings from patient records, following the same severity categorization utilized in this study. Although specific risk distributions were not disclosed, a direct proportional relationship between CONUT score and mortality rate was observed, with an AUC of 0.72 for 180-day mortality, which was slightly higher than our findings [25].

### 4.4. PNI

Similar to CONUT, PNI is based solely on blood values. This study also found that the PNI exhibited a weak predictive power for postoperative mortality. Notably, an above-average number of patients was classified as having a high nutritional risk using the thresholds established by Onodera et al. [36].

Canbolat et al. [37] published a study in 2021 assessing the preoperative PNI of 172 patients with hip fractures, finding only 53 patients (30.8%) had a concerningly low PNI. Importantly, they set a low PNI threshold of 37.25, which differed from our reference value of 40. Additionally, Canbolat et al. [37] excluded patients who underwent general anesthesia, whereas our study did not impose such criteria.

Feng et al. [38] also examined postoperative mortality related to PNI in hip surgery patients, establishing a lower threshold for malnutrition at 38 and reporting a malnutrition rate of 13.3% (26 from 263 patients). Their cohort included only patients over 70 years of age undergoing elective hip surgery, in contrast to our focus on traumatic hip fractures [38].

He et al. [39] conducted a study involving 343 patients with femoral fractures and reported an increased perioperative risk associated with a low preoperative PN. Although their study population was small, it closely resembled ours in terms of key characteristics. The most significant difference was the exclusion of patients who underwent reoperations. In their study, the cut-off for PNI was determined using the Youden Index, which was set at 42.425. ROC analysis was also performed, yielding an AUC of up to 0.77, which demonstrated a slightly better predictive performance compared to our study. However, their publication did not address the fact that a low PNI, with an average of 37.83 in patients without complications and 37.05 in those with complications, still results in a high rate of malnutrition [39].

Similarly, Shirakabe et al. [40], in their research on PNI in patients with acute heart failure, set lower cut-off values. However, Hua et al. [41] used a higher threshold in their study on breast cancer patients. This variable determination of the malnutrition cut-off appears to be a common practice across different medical specialties. It seems reasonable to assume that the threshold set by Onodera et al. [36] may be somewhat high, as suggested by the referenced studies. However, all studies have consistently shown that a lower PNI correlates with poorer prognosis and outcomes.

### 4.5. GNRI

Funahashi et al. [42] found a statistically significant correlation between low GNRI scores and 30-day postoperative mortality in hip fracture patients. Fujimoto et al. [43], observing a one-year postoperative period, reported similar findings linking lower GNRI scores to increased mortality rates. These results align with those of our study, although our analysis indicated an unusually high mortality rate among patients with very high GNRI scores, a phenomenon not documented in either study. However, it is essential to note that this effect pertained to only a small fraction of the study population. Fujimoto et al. [43] included only 108 participants and had a drastically shorter observation period of 30 days, whereas Funahashi et al. [42] analyzed 1040 patients over a more extended timeframe.

Su et al. [44] conducted a retrospective analysis of data from 678 patients over 65 years treated for femur fractures at a Taiwanese hospital over a decade. Their findings, which included both proximal and distal femoral fractures, demonstrated a significantly elevated mortality rate in patients with GNRI scores < 82, consistent with our results, although their observation period was significantly shorter. Again, a higher mortality rate associated with very high GNRI scores has not yet been reported [44].

## 5. Conclusions

The predictive power of the four nutritional scores examined in this study with regard to postoperative mortality was limited when considering the predictive models and ROC analyses employed. Although a statistically significant correlation between individual nutritional scores and postoperative mortality in our patient cohort was demonstrated, none of the scores emerged as a strong predictor of postoperative outcomes.

Based on the data at hand, no specific recommendation can be made for the implementation of a particular nutritional screening protocol for patients undergoing surgery for proximal femur fractures. Nevertheless, given the significant association between malnutrition and increased postoperative mortality, a general evaluation of nutritional status, possibly supplemented with dietary support, should be considered for this patient group.

### Limitations

The main limitation of this study stems from its retrospective design and post hoc evaluation of nutritional scores based on previously documented data. This introduces the possibility of inaccuracies or errors, particularly with regard to recent weight loss. Factors, such as water retention or the effects of diuretic treatment, may lead to greater variations in body weight, especially in elderly patients.

## Figures and Tables

**Table 1:** Mortality rates according to different postoperative (pOP) periods.

pOP-Time	Death	Mortality Rate	Death/Month
1	81	5.4%	81
3	165	11%	55
6	225	15%	37.5
12	321	21.4%	26.75
24	473	31.5%	19.7

**Table 2:** Risk ratios (RR) over time according to different nutritional scores.

pOP-Time	RR GMS ≥ 3	RR CONUT ≥ 5	RR PNI < 40	RR GNRI < 92
1	2.35	2.25	2.41	1.96
3	2.24	2.34	2.44	2.05
6	2.18	2.03	2.19	1.82
12	2.00	1.69	1.98	1.80
24	1.68	1.46	1.74	1.58

pOP-Time: postoperative-time; RR: risk ratios; CONUT: Controlling Nutritional Status Score; PNI: Prognostic Nutritional Index; GNRI: Geriatric Nutritional Risk Index.

**Table 3:** Distribution of CONUT values.

CONUT Value	n	%
0	21	1.4%
1	63	4.2%
2	124	8.3%
3	180	12%
4	244	16.3%
5	228	15.2%
6	184	12.3%
7	157	10.5%
8	129	8.6%
9	92	6.1%
10	44	2.9%
11	18	1.2%
12	16	1%

## Data Availability

The data that support the findings of this study are available on request from the corresponding author, D.P. The data are not publicly available due to restrictions containing information that could compromise the privacy of research participants.
